# Exemplar MobileNetV2-Based Artificial Intelligence for Robust and Accurate Diagnosis of Multiple Sclerosis

**DOI:** 10.3390/diagnostics13193030

**Published:** 2023-09-23

**Authors:** Tuba Ekmekyapar, Burak Taşcı

**Affiliations:** 1 Department of Neurology, Elazığ Fethi Sekin City Hospital, Elazig 23280, Turkiye; 2 Vocational School of Technical Sciences, Firat University, Elazig 23119, Turkiye

**Keywords:** multiple sclerosis, MobileNetV2, KNN, IMrMr

## Abstract

Multiple sclerosis (MS) is a chronic autoimmune disease of the central nervous system that prominently affects young adults due to its debilitating nature. The pathogenesis of the disease is focused on the inflammation and neurodegeneration processes. Inflammation is associated with relapses, while neurodegeneration emerges in the progressive stages of the disease. Magnetic resonance imaging (MRI) is commonly used for the diagnosis of MS, and guidelines such as the McDonald criteria are available. MRI is an essential tool to demonstrate the spatial distribution and changes over time in the disease. This study discusses the use of image processing techniques for the diagnosis of MS and specifically combines the MobileNetV2 network with exemplar-based learning, IMrMr feature selection, and K-Nearest Neighbors (KNN) classification methods. Experiments conducted on two different datasets (Dataset 1 and Dataset 2) demonstrate that these methods provide high accuracy in diagnosing MS. Dataset 1 comprises 128 patients with 706 MRI images, 131 MS patients with 667 MRI images, and 150 healthy control subjects with 1373 MRI images. Dataset 2 includes an MS group with 650 MRI images and a healthy control group with 676 MRI images. The results of the study include 10-fold cross-validation results performed on different image sections (Axial, Sagittal, and Hybrid) for Dataset 1. Accuracy rates of 99.76% for Axial, 99.48% for Sagittal, and 98.02% for Hybrid sections were achieved. Furthermore, 100% accuracy was achieved on Dataset 2. In conclusion, this study demonstrates the effective use of powerful image processing methods such as the MobileNetV2 network and exemplar-based learning for the diagnosis of MS. These findings suggest that these methods can be further developed in future research and offer significant potential for clinical applications in the diagnosis and monitoring of MS.

## 1. Introduction

Multiple sclerosis (MS) is a chronic autoimmune disease of the central nervous system (CNS) in which inflammation, demyelination, and axonal loss occur [1,2]. MS is the most common cause of disability in young individuals, other than trauma [3]. While it was thought to be a T-cell-mediated autoimmune disease in the past, it is now known that B cells are also effective in the pathogenesis in addition to T cells [4]. The effectiveness of drugs targeting B cells supports this idea [5]. Inflammation and neurodegeneration play a role in the pathogenesis of the disease. While inflammation has been associated with attacks, neurodegeneration has been associated with relapse-free progression [6,7]. Inflammatory infiltrates in active MS plaques are rich in CD8 (+) T lymphocytes. B cells, such as plasma cells, are less common. As a result of inflammation created by T cells, damage occurs in oligodentrocytes, and as a result, demyelination occurs. Axonal damage develops in the advanced stages of the disease. Axonal damage is irreversible [5,8,9]. Magnetic resonance imaging (MRI) in multiple sclerosis is the most sensitive radiological imaging method in demonstrating MS plaques [2]. Various guidelines have been published describing the radiological findings in the diagnosis of multiple sclerosis. McDonald’s diagnostic criteria, first published in 2001, were revised first in 2010 and then in 2017 [2]. In 2016, diagnostic criteria for magnetic resonance imaging in MS (MAGNIMS) were published [10]. According to the 2017 McDonald diagnostic criteria, which are widely used in the diagnosis of MS, at least two MS plaques must be present in the periventricular, cortical or juxtacortical, infratentorial, and spinal cord areas to meet the criteria of dissemination in space [11]. The presence of a new T2 lesion or a gadolinium (Gd)-enhancing lesion that was not in the previous MRI, or the presence of simultaneous Gd-enhancing and non-Gd-enhancing lesions in the MRI performed at any time in order to meet the criteria of dissemination in time; or the presence of an oligoclonal band in the cerebrospinal fluid (CSF) should be present in patients who meet the criteria of dissemination in space [11].

### 1.1. Literature Review

Within the realm of academic research, artificial intelligence (AI) methods have been extensively employed for the detection and diagnosis of a wide range of diseases [12,13,14,15,16]. In particular, numerous studies have harnessed deep learning (DL) and machine learning (ML) techniques to identify and diagnose multiple sclerosis (MS). fano et al. [17] conducted a cohort study of 51 MS patients and 20 healthy controls. Naïve Bayes with logistic regression models and Random decision-tree meta were utilized as classifiers. The wrapper method is implemented for feature selection, reaching 80.00% accuracy. Siar et al. [18] classified 320 MS and 791 healthy control MR images. As a preprocessing step, the spaces around the MR brain image were erased. The dataset was trained with a 25-layer CNN network. Their classification results (accuracy, sensitivity, and precision) were found to be 96.68%, 94.64%, and 100%, respectively. Eitel et al. [19] presented an individual patch filter layer-based MS classification model, and their dataset included 76 MS and 71 healthy control MR images. An 80.92% accuracy was achieved. Alijamaat et al. [20] applied histogram stretching and data augmentation preprocessing to MR images from 38 MS and 20 healthy controls. Their proposed method used a two-dimensional discrete Haar wavelet transform with CNN network. Accuracy, sensitivity, and precision results of 99.05%, 99.14%, and 98.43%, respectively, were obtained. Sepehvand et al. [21] classified MS disease with a 3D CNN network. The dataset in their study included 1068 MS patients. In their proposed end-to-end 3D CNN network, accuracy, specificity, sensitivity, and precision results of 80.21%, 79.16%, 80.11%, and 91.82% were obtained, respectively. Narayana et al. [22] detected deep learning-based MS using 1970 MR images from 1008 patients. Anisotropic diffusion filtering, skull stripping, and bias correction were applied as pre-treatment measures. In their proposed method using the VGG16 network, they obtained a specificity of 72.00% and sensitivity of 70.00%. Krishnamoorthy et al. [23] detected MS lesions with the CNN network. The dataset included 1000 healthy controls and 1000 MS images. The authors compared AlexNet, VGG16, VGG19, ResNet18, and ResNet50 pre-trained models. With VGG16, accuracy, specificity, sensitivity, and precision results of 94.50%, 94.06%, 94.95%, and 94.00% were obtained, respectively. Zhang et al. [24] classified MS by changing the layers of the AlexNet network. The dataset included the MR images of 20 healthy controls and 38 MS patients, obtaining an accuracy of 98.17%, specificity of 98.21%, and F1-score of 98.15%.Zhuo et al. [25] classified astrocytoma, ependymoma, MS, and neuromyelitis optica spectrum disorders using MultiResUNet and DenseNet121 networks. The dataset included images of 490 patients, and achieved an accuracy of 96%.Caba et al. [26] classified MR images for detecting MS. There were 1512 images of MS patients in the ADVANCE dataset, 886 patients with MS in the ASCEND dataset, and 1841 images of patients with MS in the DECIDE dataset. In their proposed CNN model, an 82.4% accuracy was obtained with the α-radiomics classifier. Acar et al. [27] diagnosed MS using the 3D discrete wavelet transform method from 3D MR imagery. The dataset consisted of 3D MR images from 40 patients. An F1-score of 95.00% was obtained for precision and recall.

### 1.2. Novelties and Contributions

#### 1.2.1. Novelties

In terms of innovations, the MobileNetV2 [28] model brings significant advancements to the table. This model adopts a novel approach to deep feature extraction, distinguishing itself from traditional deep learning models. By using innovative convolutional layers such as Depthwise Separable Convolution, it offers a more efficient feature extraction process compared to traditional CNN architectures. This results in a model that can operate faster with lower computational requirements. Additionally, pre-trained MobileNetV2 models are trained on a large dataset, providing a suitable foundation for transfer learning applications, enabling the model to be used quickly and effectively in various visual recognition tasks.

#### 1.2.2. Contributions

This study introduces a customized approach to the analysis of magnetic resonance imaging (MRI) data, making significant contributions. Firstly, we developed a data processing method by breaking down the 224 × 224 pixel-sized MRI images into smaller 28 × 28 pixel-sized patches. This allowed for more efficient processing of image data and reduced processing times. Subsequently, we devised an original feature extraction strategy using the MobileNetV2 architecture to process the data obtained from these 28 × 28 pixel-sized patches. This strategy focused on extracting unique features rather than fully connected layers and resulted in a total of 65,000 features being extracted. The main advantages of this method are its ability to reduce data size while preserving important features. As a result, this study offers an effective approach for feature extraction on MRI images, potentially enhancing its usability in medical image analysis and diagnostic applications. It may also provide guidance for future research. This study contributes to the field by presenting a new method for feature extraction from MRI images, advancing the field’s development.

### 1.3. Motivation and Proposed Method

MS is a chronic inflammatory disease of the central nervous system, affecting millions of individuals worldwide, presenting a significant global health concern [2,3]. The diagnosis and ongoing monitoring of MS are of critical importance in enhancing patients’ quality of life and improving treatment outcomes. Traditionally, the diagnosis and monitoring of MS have relied upon expert neurologists’ clinical assessments, medical imaging such as magnetic resonance imaging (MRI), and clinical tests [2]. However, these methods can be subjective, time consuming, and susceptible to human errors. In recent years, it has become increasingly evident that artificial intelligence (AI) and deep learning techniques hold substantial potential in the realm of medical image analysis and diagnosis. Specifically, exemplar-based AI models offer a novel approach for detecting and monitoring complex neurological diseases by utilizing extensive datasets. The primary motivation of this article is to explore the potential utilization of AI-based exemplar models for the diagnosis and monitoring of MS.

### 1.4. Organization

The remaining organization of this research is outlined below. In Section 2, we present the Materials and Methods, where we describe the proposed model. Section 3 will examine the experimental results. In Section 4, we will discuss the findings and conclusions. Finally, in Section 5, we will conclude our research.

## 2. Materials and Methods

### 2.1. Preprocessing

**Figure 1:**
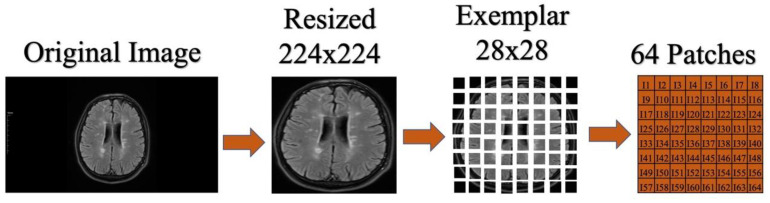
Preprocessing applied to MR images.

In this section, we will explain the significant steps undertaken to process raw brain images. Firstly, a procedure called “brain segment area” was applied to the brain images to ensure they only contained the brain region. This process effectively retained information related solely to the brain in the processed images. Subsequently, these cropped images were resized to 224 × 224 pixels. Resizing the images to this dimension made them more suitable for use during processing. Finally, these 224 × 224 sized images were equally divided into 28 × 28 pixel dimensions. This division allowed the images to be broken down into smaller, manageable parts and made them suitable for subsequent analyses. These preprocessing steps are fundamental operations applied to convert raw brain images into more manageable and meaningful data for further processing(see Figure 1).

### 2.2. Proposed Method

In this study, we propose the utilization of an exemplar-based artificial intelligence model to detect the diagnosis of multiple sclerosis (MS). Our research involved working with two distinct datasets. The suggested model is based on deep learning methods and utilizes the MobileNetV2 model as its foundation. This model consists of six stages: (i) In the first stage, preprocessing is applied to the images in the dataset. (ii) Subsequently, end-to-end training is conducted on the dataset using the MobileNetV2 model. (iii) Images in the dataset are transformed into exemplar images of size 28 × 28, and fully connected layers are used to extract features for each exemplar. (iv) The 65,000 features extracted from 64 exemplar images of size 28 × 28 and the original images are merged. (v) The merged features are selected using an iterative feature selector. (vi) The selected features are classified using the K-Nearest Neighbors (KNN) [29] algorithm with 10-fold cross-validation.

**Figure 2:**
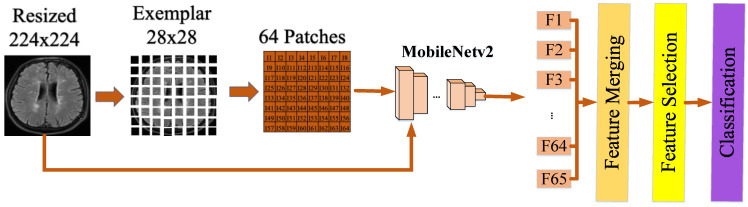
Proposed model.

The graphical representation of the proposed MobileNetV2 exemplar model is presented in Figure 2.

The fundamental steps of the proposed model are tabulated in Table 1.

This step is used to obtain the results of the proposed model and diagnose multiple sclerosis.

These steps collectively form the core of the proposed model, which is designed for the diagnosis of multiple sclerosis.

### 2.3. Dataset

In this study, two different datasets were utilized. The first dataset is the Multiple Sclerosis (MS) Magnetic Resonance Imaging (MRI) dataset found on Kaggle [30]. The second dataset is the MS dataset collected by the eHealth Laboratory at Cyprus University [31]. The Kaggle MS MRI dataset comprises four different data categories: MS-Axial, MS-Sagittal, Healthy-Axial, and Healthy-Sagittal groups. Both MS-Axial and MS-Sagittal groups consist of individuals with multiple sclerosis (MS), with a total of 72 participants, including 21 males and 51 females. The average age for both MS groups is determined to be 28.4 ± 5.66 years and is categorized based on MRI imaging methods. Specifically, the MS-Axial group contains 650 MRI images, while the MS-Sagittal group contains 761 MRI images. On the other hand, the Healthy-Axial and Healthy-Sagittal groups consist of healthy individuals, with a total of 57 and 49 participants, respectively. The average ages for the healthy individual groups are 29.5 ± 8.32 years and 27.4 ± 6.48 years, and the number of MRI images in each group is specified. The Healthy-Axial group contains 1002 MRI images, while the Healthy-Sagittal group contains 1014 MRI images.

**Figure 3:**
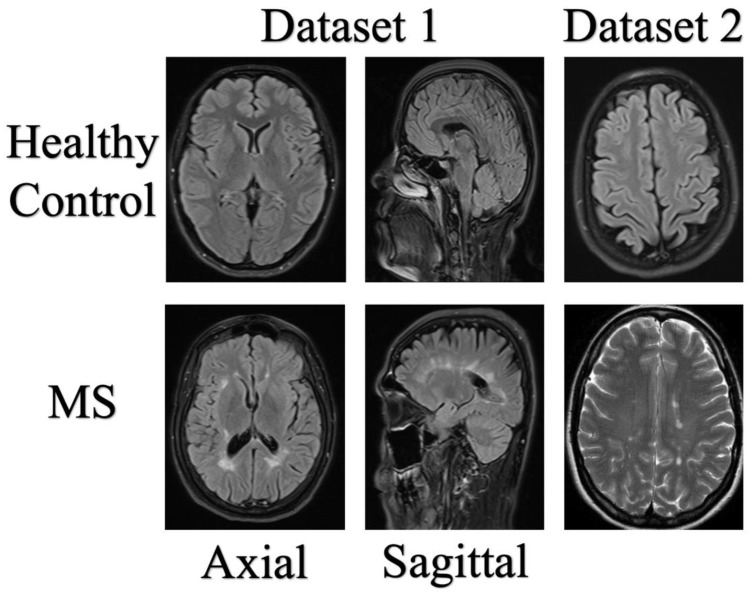
Sample images in datasets.

The MS dataset collected by the eHealth Laboratory at Cyprus University consists of a total of 676 MRI images from 38 MS patients. The average age of these 38 patients is 34.1 years, with 17 males and 21 females among them. The sample images from the dataset we used are shown in Figure 3.

### 2.4. MobileNetv2

MobileNet is a convolutional neural network developed by the Google team in 2017. MobileNet is designed to be lightweight, particularly in terms of size and computational requirements, while still aiming to deliver high performance. MobileNetV2 [28], on the other hand, is the second version of MobileNet and features fewer parameters compared to its predecessor. This reduction in parameters enables the creation of lighter and more efficient deep learning models. Its lightweight architecture, optimized for mobile devices and embedded systems, makes it an ideal solution for high-performance image processing applications in such devices. MobileNetV2 can also be used as a pre-trained model. Pre-trained models are deep neural networks trained on extensive image datasets and can subsequently be fine-tuned for various tasks. MobileNetV2 simplifies the image processing pipeline and helps transform image data into a format that the model can comprehend, leading to more accurate results. The linear bottleneck layer of the model accelerates learning and enhances model stability by using the ReLU activation function. This contributes to MobileNetV2’s ability to learn faster and achieve better results. Consequently, MobileNetV2 stands out as an ideal choice for mobile devices and embedded systems due to its lightweight design and high performance.

### 2.5. Classification

The K-Nearest Neighbors (KNN) classifier fundamentally uses the class labels of neighboring examples to classify a given sample. This classifier employs a distance metric to compute similarity between examples and utilizes the class labels of the K-Nearest Neighbors to classify an instance. KNN is known for its simple and interpretable structure and has been successfully applied in various domains. However, for it to work effectively, determining the correct value of K and other hyperparameters is necessary. Here are the results we obtained using the KNN classifier in our experiments:

- The accuracy rate achieved on the validation data was 99.8%.
- The total cost (validation) was 4.
- The prediction speed was approximately 170 observations per second.
- The training time took 149.34 s.
- The model size in its compact form was approximately 17 MB.

Let us delve into the key hyperparameters and settings used:

1. Preset (setting): The KNN classifier has a specific preset, which determines the type of KNN algorithm to be used. In this study, the “Subspace KNN” preset was utilized. Subspace KNN applies the KNN algorithm using a subset of features, allowing for effective feature selection and contributing to better model performance.
2. Ensemble method: The KNN classifier operates using an ensemble method. Ensemble methods aim to create a stronger and more robust model by combining multiple learners.
3. Learner type: In this study, the learner type chosen is “Nearest Neighbors”. KNN classifies an instance based on the class labels of its nearest neighbors, relying on neighborhood relationships in the data for classification.
4. Number of learners: The model operates as an ensemble, consisting of 30 learners. This allows the model to create a more powerful classifier by combining multiple learners.
5. Subspace dimension: The dimension of the subspace used was set to 86. The subspace dimension determines which subset of features will be utilized. This is an important hyperparameter in feature engineering and feature selection processes.

These hyperparameters help optimize the KNN classifier for a specific application and contribute to achieving high accuracy and low cost in this study.

### 2.6. Feature Selection with Iterative MRMR (Minimum Redundancy Maximum Relevance)

Minimum Redundancy Maximum Relevance (MRMR) is an important method in the field of feature selection, offering several advantages in the process. Firstly, MRMR carefully selects features from the dataset to enhance the performance of a classification model. The chosen features are highly relevant to the classification task, leading to improved classification results. Secondly, MRMR adheres to the principle of minimum redundancy, minimizing similarities among the selected features, thereby enhancing the overall performance of the model. Thirdly, MRMR can reduce data dimensionality, potentially speeding up the model while saving computational power and time. Iterative Minimum Redundancy Maximum Relevance (iMRMR) [32] is an extended or iterative version of the traditional MRMR (Minimum Redundancy Maximum Relevance) feature selection algorithm. iMRMR is designed to perform feature selection with greater flexibility and precision. It employs an iterative approach, where initially, a base feature set is chosen, and then this set is gradually refined. During iterations, features are added or removed, and MRMR criteria are recalculated at each step. This process continues until a better feature set is found. iMRMR starts by ranking features and then selects or removes features based on the ranking results, providing more control and customization options.

## 3. Experimental Results

In this study, the effectiveness of the MobileNetV2-based exemplar deep feature extraction approach was verified using two different multiple sclerosis (MS) magnetic resonance imaging (MRI) datasets. The MobileNetV2-based exemplar deep feature extraction model was implemented using MATLAB (R2023a) software. High-performance desktop computer hardware was used for the analysis, equipped with an Intel(R) Core(TM) i9-13900 CPU running at 5.8 GHz, 128 GB of RAM, and a Windows 11 operating system. MATLAB (.m) files were used for the proposed pyramid deep feature extraction and processing of MR image data. The obtained features were then processed for classification using the Classification Learner App Toolbox. We did not use any hyperparameter optimization model. We used the default parameters of the machine learning methods.To achieve the best classification results, the Subspace K-Nearest Neighbors (KNN) classification algorithm was preferred.

**Figure 4:**
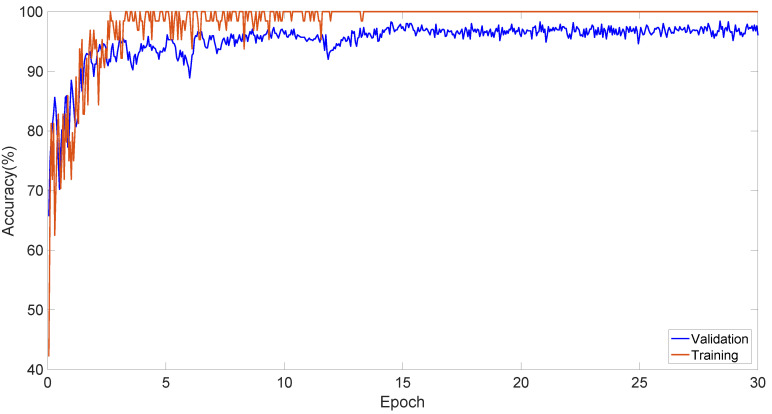
The training and validation performance curves.

In Dataset 1, Axial and Sagittal images were combined and trained with the MobileNetV2 model. During training, the data were split into 70:30 for training and validation. The training and validation performance curves for these datasets are shown in Figure 4.

**Figure 5:**
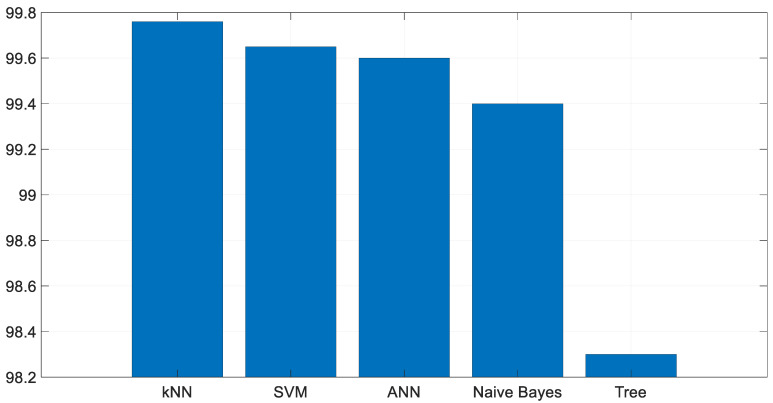
Different classifier results.

In Figure 3, the Axial and Sagittal images were used to train the MobileNetV2 network from end to end. This trained MobileNetV2 network was then used for the proposed exemplar model. To decide which classifier to use in the study, the features extracted from Axial images were classified using K-Nearest Neighbors (KNN), Support Vector Machine (SVM), Artificial Neural Network (ANN), Naive Bayes, and Decision Tree classifiers (see Figure 5). Among these classifiers, KNN provided the highest accuracy, which is why it was chosen for use in this study.

**Figure 6:**
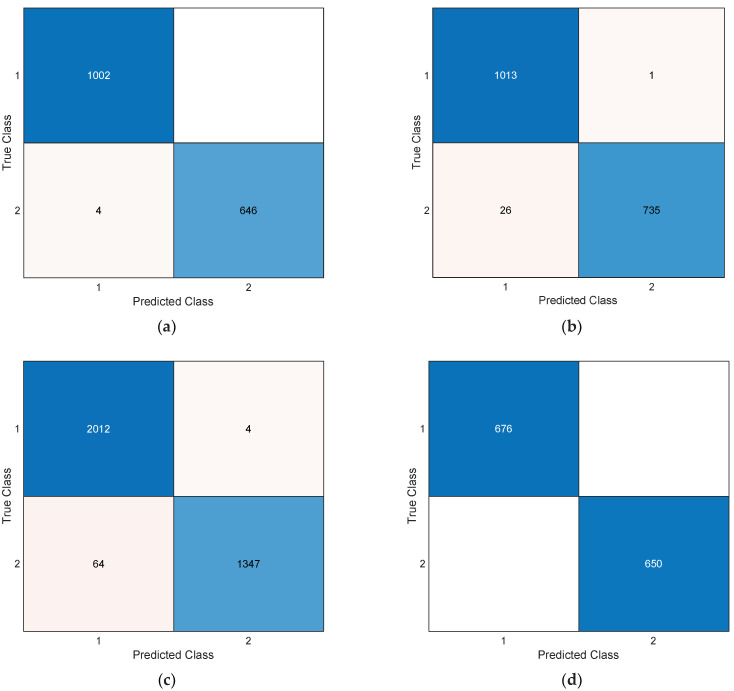
Confusion matrices of the used dataset. (**a**) Dataset 1Axial, (**b**) Dataset 1 Sagittal, (**c**) Dataset 1 Hybrid, (**d**) Dataset 2. (1) Healthy control, (2) MS.

Performance metrics are crucial criteria used to assess the success of a model or algorithm. In this study, performance metrics including accuracy, F1 score, recall, and precision were calculated using the confusion matrices in Figure 6 (see Table 2).

This study aimed to evaluate the performance of an image processing-based method for the diagnosis of multiple sclerosis (MS). Using two different datasets, a series of metrics were employed to measure the ability to distinguish between healthy controls and MS patients. Here is a summary of our experimental results:

- In Dataset 1 Axial view, high accuracy was achieved for healthy controls and MS classes. The accuracy rate for the healthy control group was 99.76%, while for the MS group, it was 99.69%. This indicates that the method accurately classified non-MS individuals.
- In Dataset 1 Sagittal view, high accuracy and sensitivity were obtained for healthy controls and MS classes. The accuracy rate for the healthy control group was 99.48%, and the sensitivity was 97.50%. For the MS group, the accuracy was 98.20%, and the recall was 99.86%.
- In Dataset 1 Hybrid view, high accuracy and sensitivity were also observed for healthy controls and MS classes. The accuracy for the healthy control group was 98.02%, sensitivity was 96.92%, and recall was 99.80%.

These results demonstrate that the image processing-based approach exhibited high performance in the diagnosis of MS. Specifically, it is noted that the Axial view provided high sensitivity, while the sagittal view stood out with high recall.

- Dataset 2 exhibited perfect performance for both classes (healthy controls and MS). Accuracy, sensitivity, recall, and F1-score were all calculated as 100% for both classes. This indicates that the model was perfectly discriminating in this dataset.

In conclusion, this study suggests that an image processing-based method could be a potentially valuable tool for the diagnosis of MS. The obtained high accuracy, sensitivity, and recall values indicate that this approach should be considered for clinical applications. Additionally, it should be noted that the combination of different view types can be used to achieve better results. In the future, further validation on larger patient groups and preparation for clinical use will be necessary for this method.

## 4. Discussion

This study aimed to improve the detection of multiple sclerosis (MS) using MRI images. To achieve this goal, a pre-trained deep learning network named MobileNetV2 was chosen. Additionally, the use of a pre-trained model helped us to learn the data more quickly and efficiently. Another significant aspect of this study is the end-to-end training of MobileNetV2 on MS images. This approach is intended to optimize the model’s ability to detect MS. Consequently, it allowed the model to better adapt to the characteristics of the data and effectively identify the disease. Furthermore, the use of exemplar images is a fundamental part of this study. Exemplar images were included to support and optimize the model’s learning process. These images helped the model gain a better understanding of MS and distinguish it, thus enhancing the overall performance of the model (see Table 3).

Macin et al. [30] present a model that utilizes methods such as ExMPLPQ, INCA, and KNN. It was tested on 1411 MRI data with 10-fold cross-validation. The results indicate high accuracy, sensitivity, and specificity values for Axial, Sagittal, and Hybrid imaging approaches. Tatli et al. [33] used a model named MSNet. It was tested on 706 MRI data and achieved high accuracy, sensitivity, and F1-scores based on 10-fold cross-validation results. Wang et al. (2021) [34] employed a six-layer CNN and stochastic pooling. It was tested on 676 MRI data and achieved high accuracy, sensitivity, and F1-scores. However, these results are slightly lower compared to other studies. Alijamaat et al. (2021) [35] utilized CNN and data augmentation techniques. It was tested on 676 MRI data and achieved high accuracy, sensitivity, and specificity.

The limitations of the exemplar MobileNetV2 model are given below.

Limitations:

- This study utilized brain MRI images from 144 healthy individuals and 106 patients with multiple sclerosis (MS). However, in order to achieve more reliable results and develop a generalizable model, it is necessary to test the model with a more diverse and varied dataset.
- Feature extraction from patches is a time-consuming process.
- In this study, we cannot determine the precise location of MS lesions, as MS lesions can sometimes be found in multiple regions. To address this issue, the use of an intelligent segmentation model may be required.

## 5. Conclusions

MS is a chronic autoimmune disease of the central nervous system and is one of the most common disabling factors in young individuals, often occurring after trauma. The diagnosis and monitoring of MS rely on clinical assessments, magnetic resonance imaging, and clinical tests. However, these methods can be subjective and timeconsuming. In recent years, artificial intelligence and deep learning techniques have introduced a new approach to the analysis and diagnosis of medical images. The aim of this study is to investigate the potential of an artificial intelligence-based model for the diagnosis of MS. The results of the research demonstrate that a specially developed MobileNetV2-based model can successfully distinguish between healthy control groups and MS patients. The accuracy results obtained for Dataset 1 Axial, Dataset 1 Sagittal, Dataset 1 Hybrid, and Dataset 2 are 99.76%, 99.48%, 98.02%, and 100%, respectively. High accuracy, sensitivity, recall, and F1-score values support the usability of this model in clinical applications. Additionally, it has been observed that different image views (Axial, Coronal, and Sagittal) yield different performance results. In conclusion, this study suggests that an artificial intelligence-based approach may be effective for the analysis of magnetic resonance image data. In the future, further studies can be conducted using larger datasets and more complex deep learning models. Additionally, more testing and validation are required to assess the effectiveness of this approach in clinical applications.

## Figures and Tables

**Table 1:** Fundamental steps.

Step	Description
1	Train Sagittal and Axial images of Dataset I using the MobileNetV2 model for foundational training.
2	Resize each image to 224 × 224 pixels and divide them into smaller 28 × 28-pixel dimensions for manageable processing.
3	Process each part of the images and the main image using the fully connected layer of MobileNetV2 to extract 1000 features from each.
4	Merge the extracted features, resulting in a total of 65,000 features, creating a comprehensive feature set.
5	Select the most informative and meaningful features using the ImRMR (Minimum Redundancy Maximum Relevance) feature selector.
6	Perform classification with 10-fold cross-validation (CV) using the selected features and the k-Nearest Neighbors (KNN) classifier to diagnose multiple sclerosis.

**Table 2:** Performance metric results.

Dataset	Class	Accuracy (%)	F1-Score (%)	Recall (%)	Precision (%)
Dataset 1 Axial	Healthy control	99.76	99.80	100	99.60
	MS		99.69	99.38	100
Dataset 1 Sagittal	Healthy control	99.48	98.68	99.90	97.50
	MS		98.20	96.58	99.86
Dataset 1 Hybrid	Healthy control	98.02	98.34	99.80	96.92
	MS		97.54	95.46	99.70
Dataset 2	Healthy control	100	100	100	100
	MS		100	100	100

**Table 3:** Comparison of studies in the literature.

Study	Method	Dataset	Split: Ratio	Performance Metrics (%)
**Macin et al. (2022)** [30]	ExMPLPQ, INCA, KNN	MS 1411 MRI, 144 patients Healthy control 2016 MRI, 106 subjects	10-fold CV	Axial: Accuracy: 98.37, Sensitivity: 96.46, Specificity: 99.60 Sagittal: Accuracy: 97.75, Sensitivity: 95.01. Specificity: 99.80 Hybrid: Accuracy: 98.22, Sensitivity: 96.39, Specificity: 99.50
**Tatli et al. (2023) [33]**	MSNet: DenseNet201, ResNet50, NCA, RF, Chi2, SVM, KNN	MS 706 MRI, 128 patients Myelitis 667 MRI, 131 patients Healthy control 1373 MRI, 150 subjects	10-fold CV	Accuracy: 97.13 Precision:97.23 Recall:97.22 F1-score:97.23
**Wang et al. (2021)** [34]	Differentiate normalization, expanding histogram range, employing a six-layer CNN alongside stochastic pooling.	MS 676 MRI, 28 patients Healthy control 681 MRI, 26 subjects	10-fold CV	Accuracy: 95.82 Sensitivity: 95.98 Specificity: 95.67 Precision: 95.66 F1-Score: 95.81
**Alijamaat et al. (2021)** [35]	A rotation range of 30 degrees, a shear conversion percentage of 0.2, a zoom range of 0.1, and the utilization of a CNN featuring wavelet pooling.	MS 676 MRI, 38 patients Healthy control 615 MRI, 20 subjects	80:20	Accuracy: 98.92 Sensitivity: 99.20 Specificity: 98.33 Precision: 99.20
**Our model**	Exemplar MobileNetv2, IMrMr, kNN	Dataset 1 MS1411 MRI, 144 patients Healthy control 2016 MRI, 106 subjects Dataset 2 650 MRIMS 676 MRIhealthy control	10-fold CV	Dataset 1 Axial: Accuracy: 99.76 Dataset 1 Sagittal: Accuracy: 99.48 Dataset 1 Hybrid: Accuracy: 98.02 Dataset2 Accuracy: 100

## Data Availability

In this paper, the dataset is publicly available.
